# LSD1 mediates MYCN control of epithelial-mesenchymal transition through silencing of metastatic suppressor NDRG1 gene

**DOI:** 10.18632/oncotarget.12924

**Published:** 2016-11-22

**Authors:** Susanna Ambrosio, Stefano Amente, Carmen D. Saccà, Mario Capasso, Raffaele A. Calogero, Luigi Lania, Barbara Majello

**Affiliations:** ^1^ Department of Biology, University of Naples ‘Federico II’, Naples, Italy; ^2^ Department of Molecular Medicine and Medical Biotechnologies, University of Naples, ‘Federico II’, Naples, Italy; ^3^ CEINGE Biotecnologie Avanzate, Napoli, Italy; ^4^ Molecular Biotechnology Center, Department of Molecular Biotechnology and Health Sciences, University of Torino, Turin, Italy

**Keywords:** MYCN, LSD1, NDRG1, EMT, neuroblastoma

## Abstract

Neuroblastoma (NB) with MYCN amplification is a highly aggressive and metastatic tumor in children. The high recurrence rate and resistance of NB cells to drugs urgently demands a better therapy for this disease. We have recently found that MYCN interacts with the lysine-specific demethylase 1 (LSD1), a histone modifier that participates in key aspects of gene transcription. In cancer cells, LSD1 contributes to the genetic reprogramming that underlies to Epithelial-Mesenchymal Transition (EMT) and tumor metastasis. Here, we show that LSD1 affects motility and invasiveness of NB cells by modulating the transcription of the metastasis suppressor NDRG1 (N-Myc Downstream-Regulated Gene 1). At mechanistic level, we found that LSD1 co-localizes with MYCN at the promoter region of the NDRG1 gene and inhibits its expression. Pharmacological inhibition of LSD1 relieves repression of NDRG1 by MYCN and affects motility and invasiveness of NB cells. These effects were reversed by overexpressing NDRG1. In NB tissues, high levels of LSD1 correlate with low levels of NDRG1 and reduced patients survival. Collectively, our findings elucidate a mechanism of how MYCN/LSD1 control motility and invasiveness of NB cells through transcription regulation of NDRG1 expression and suggest that pharmacological targeting of LSD1 represents a valuable approach for NB therapy.

## INTRODUCTION

Neuroblastoma (NB), a disease of the sympathetic nervous system, is the most common solid tumor of infancy. Despite significant advances in the treatment of pediatric cancer over the past two decades, NB remains a highly refractory malignancy, with less than 50% 5-year survival rates for the majority of patients who are diagnosed with high-risk disease. One of the most powerful independent prognostic indicators for this disease is the amplification of the MYCN oncogene, which occurs at high levels in approximately 25% of NBs [1–3]. High-risk NBs often present hematogenous metastasis indicating that MYCN amplification control epithelial-mesenchymal transition (EMT) through which NB cells lose homotypic adhesion and acquire migratory capacity [4]. High level of MYCN expression has a great impact on global gene expression. [5]. Despite this richness of information, the entire and precise network of interactions that MYCN establishes within cancer cells remains elusive. Recently, we have demonstrated that MYCN interacts with LSD1/KDM1A, a monoamine oxidase that function as master epigenetic regulator in NB cell lines and that the MYCN/LSD1 complex is involved either in activation or repression of MYCN target genes in NB cell lines [6]. Importantly, the inhibition of LSD1 activity reduces neuroblastoma cell viability and induces differentiation. These findings suggest that LSD1 inhibition may have strong therapeutic relevance to counteract MYCN-driven oncogenesis.

LSD1 is an amine oxidase that catalyzes lysine demethylation in a flavin adenine dinucleotide (FAD)-dependent oxidative reaction. LSD1 removes mono- and dimethyl groups from lysine 4 (H3K4) and lysine 9 (H3K9) of histone H3, and can also targets non-histone proteins such as p53, E2F1, and DNMT1 [7–9]. LSD1 was initially described as a cofactor of the REST/CoREST complex. Although LSD1 can function as a co-repressor of transcription factors as REST, it also has been reported to function as a coactivator of specific transcription factors by removing H3K9 methylation, suggesting that its substrate specificity defines its biological outcome [10–12]. LSD1 is overexpressed in a variety of cancers and tends to correlate with more aggressive cancers with poor prognosis. There is a large body of evidence that LSD1 is involved in maintaining the undifferentiated, malignant phenotype of neuroblastoma cells and that its overexpression correlates with aggressive disease, poor differentiation and infaust outcome [13, 14].

To address the functional significance of LSD1 inhibition in NB we performed global transcriptome analysis (RNA-seq) in LSD1-deficient NB cells. Analysis of differentially expressed gene (DEG) highlighted the biological relevance of co-target genes indicating that epithelial-mesenchymal transition pathway was significantly affected. Among genes positively affected by LSD1 inhibition we focused our attention on the metastatic tumor suppressor gene N-myc downstream regulated1, NDRG1. In fact, we find that NDRG1 is inhibited by LSD1. NDRG1 is one of the four members of the human NDRG family, and its designation comes from its expression being repressed by MYC and MYCN [15, 16] and its expression is negatively correlated with tumor progression in multiple neoplasms. NDRG1 is a potent metastatic suppressor that has been shown to restrain TGF-ß-induced EMT in prostate and colon cancer cells, while its reduction induces EMT [17–22]. Collectively these studies demonstrated that NDRG1 functions as a metastatic suppressor that inhibits EMT in human cancer a key initial step in metastasis.

We found that LSD1 inhibition suffices to de-repress NDRG1 expression even in the presence of MYCN amplification. Expression of NDRG1 suppresses motility and invasiveness of NB cells. In silico studies of neuroblastoma tumor samples revealed that low expression of NDRG1 was associated with poor survival. Low NDRG1 and high LSD1 levels were mutually exclusive in MYCN-amplified NB samples, corroborating the *in vitro* results. Taken together, our findings provide a previously unidentified model to control of EMT in NB, suggesting that LSD1 represents a novel and promising target for selective inhibition of cell migration and invasiveness in neuroblastoma cells.

## RESULTS

### LSD1 depletion selectively affects EMT pathway

**Figure 1:**
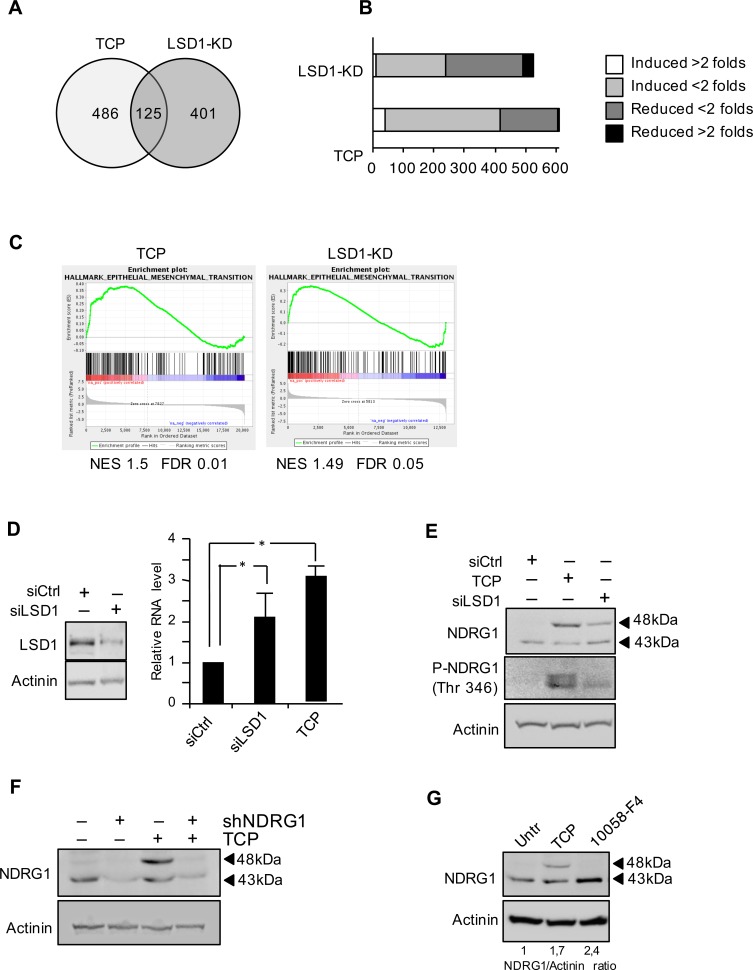
A. Venn diagram of the DEG present in both LSD1-knockdown (LSD1-KD) and TCP treatment **B**. Gene set of regulated genes by TCP treatment and LSD1-KD. **C**. Gene set enrichment analysis (GESA) plots show enrichment of gene sets regulated by LSD1-KD and TCP treatment. In each panel, nominal NES and false discovery rates (FDRs) are indicated. **D**. NDRG1 gene expression was analyzed by qRT-PCR, using samples prepared from Tet-21/N cells and treated with TCP or siRNA-LSD1 and siRNA-control as indicated. LSD1 protein level in Tet-21/N cells transfected with siRNA-LSD1 or control was determined by western blot. *, statistical significance (*P* < 0.01; Student t test). **E**. Western blotting of protein extracts from Tet-21/N cells prepared as described in D, using NDRG1 and phospho-NDRG1 (Thr 346) antibodies. **F**. NDRG1 silencing using sh-NDRG1 in Tet-21/N cells treated with TCP or vehicle, was assayed by western blot. **G**. Western blotting of protein extract from Tet-21/N treated with vehicle, TCP or 10058-F4 for 48 hrs, using NDRG1 antibody. Actinin has been probed as loading control.

LSD1 is highly expressed in undifferentiated Neuroblastoma and its high expression correlates with adverse outcome [13, 14]. We recently showed that MYCN interacts with LSD1 and that the LSD1/MYCN complex controls transcription of tumor suppressor genes such as p21 and CLU [6]. Moreover LSD1 inhibition results in cell growth arrest of cultured NB cells. To address in more details the role of LSD1 function in NB cells, we performed global transcriptome analysis (RNA-seq) of Tet-21/N cells treated with tranylcypromine (TCP) a potent inhibitor of LSD1. In parallel, we performed RNA-seq from Tet-21/N cells treated with siRNA targeting LSD1 (LSD1-KD). RNA-seq data from duplicate biological replicas were then analyzed for differentially expressed gene (DEG). Statistical analysis allows us to screen out 661 DEGs in TCP sample (log2FC ≥ 1; FDR ≤ 0.1) and 526 DEGs in LSD1-KD (log2FC ≥ 1; FDR ≤ 0.1). 125 were commonly present in both treatments (Figure 1A, 1B and Supplementary Table 3). To clarify the biological relevance of co-target genes we used Gene set enrichment analysis. GSEA revealed that among top scoring pathways the gene set of Epithelial-Mesenchymal Transition, EMT, was ranked as significantly affected in both TCP and LSD1-KD samples (Figure 1C). We quantified expression levels of EMT marker genes in TCP treated or LSD1-KD Tet-21/N cells *versus* control cells by qRT-PCR. As shown in Figure Supplementary 1, LSD1 inhibition increased the levels of the epithelial markers, E-cadherin, occludin and desmoplakin, and reduced the expression mesenchymal markers, Vimentin and α-SMA, whereas no significant differences were detected in N-cadherin expression.

Previous studies have shown that LSD1 is indeed involved in the control of EMT, through interaction with the SNAG domain of SNAI1, a master EMT regulator [23, 24]. Among the several genes that were affected in TCP-treated and LSD1-KD cells related to EMT (SAT1, PLAUR, TNFRSF12A, RGS4, BDNF, MPP3, NDRG1 and SGK1) we focused our attention on the MYCN regulated gene, the metastasis suppressor gene NDRG1 (N-myc downstream regulated gene 1). NDRG1 was first isolated as a gene up-regulated in N-Myc knockout mouse embryos [25] and directly repressed by MYCN and c-MYC through binding to the NDRG1 core promoter [26]. The metastasis suppressor NDRG1 is negatively correlated with tumor progression of several types of cancer, and most importantly down-regulation of NDRG1 expression enhances cell proliferation and invasiveness. In contrast, its up-regulation reduces cell proliferation and invasiveness [27–29].

To validate the role of LSD1 in NDRG1 expression we inhibited LSD1 in Tet-21/N cells with TCP or siRNA-targeted knockdown and measured NDRG1 mRNA and protein expression levels. We found that TCP treatment or LSD1 silencing stimulates NDRG1 expression (Figure 1D and 1E). Previously immunoblotting studies revealed that NDRG1 might appear as multiple protein bands depending from the cellular context likely due to different isoforms and/or post-translational modifications such as phosphorylation and glycosylation [27, 30, 31]. It has been shown that the signal cascade mTORC2/serum glucocorticoid induced protein kinase1 (SGK1) phosphorylates NDRG1 at T346 and this modification is essential to suppress tumor growth [20, 32]. Tet-21/N cells treated with TCP or siLSD1 were probed with an antibody that specifically recognize NDRG1 phosphorylated at T346 demonstrating that LSD1 inhibition induces NDRG1 phosphorylation, Figure 1E. Finally shRNA-targeted NDRG1 knockdown demonstrates specificity of NDRG1 bands (Figure 1F). To address the contribution of MYC and LSD1 to NDRG1 expression, Tet-21/N cells were treated with 10058-F4, a small molecule inhibitor of MYC/MAX dimerization [6] that has effect on either cMYC then MYCN. Following 10058-F4 treatment we found an increase of the 43kDa NDRG1 band, while TCP activates the 48kDa (Figure 1G). These findings suggest that inhibition of either MYCN or LSD1 de-repress NDRG1 expression. However, while MYCN inhibition activates NDRG1, LSD1-KD also induces NDRG1 phosphorylation.

### MYCN and LSD1 co-localize at NDRG1 promoter and repress its expression

**Figure 2:**
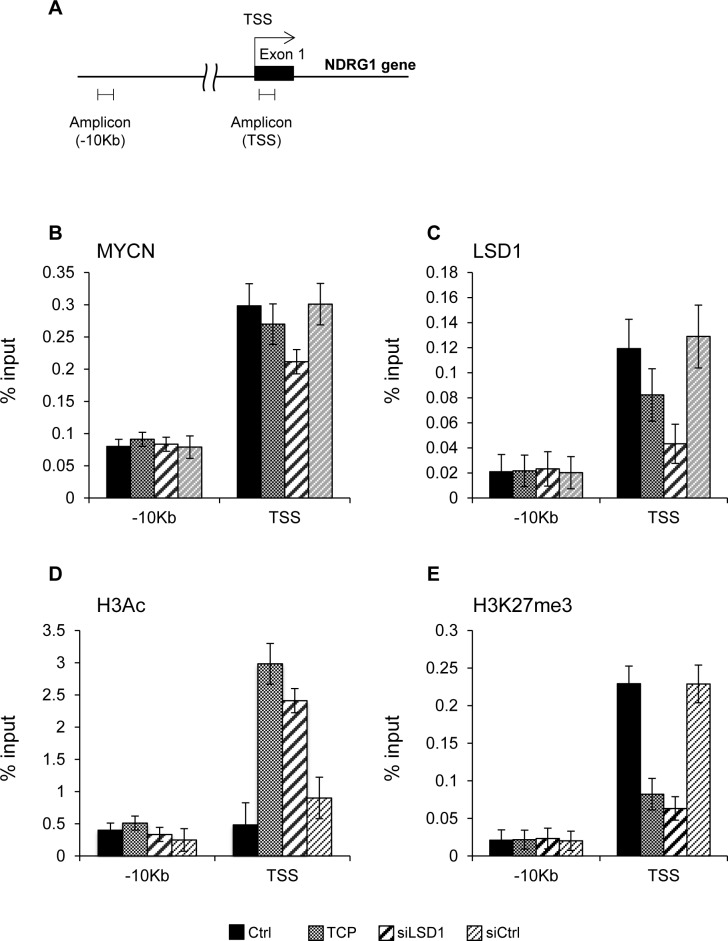
A. Schematic representation of the NDRG1 promoter **B**. and **C**. MYCN and LSD1 binding to NDRG1 chromatin. Cell treatments are indicated at the bottom of the figure. qPCR was performed with primers for NDRG1 TSS, and -10kb. **D**. and **E**. Histone modifications at NDRG1 chromatin; ChIPs were carried out using the indicated antibodies and analyzed with primers encompassing the TSS region and -10kb from TSS. Values from three independent ChIP assays are presented along with standard deviations, *n* = 3. Changes in % input are shown normalized over IgG controls and are all statistically significant (*P* < 0,05; Student t test).

To determine whether LSD1 is directly involved in transcriptional control of NDRG1 we inhibited LSD1 in Tet-21/N cells with TCP or with siRNA against LSD1 and assessed the relative binding of MYCN and LSD1 to the NDRG1 gene by chromatin immune-precipitation (ChIP) assays. The immunoprecipitated chromatin samples were subjected to qPCR using primers corresponding to the transcriptional start site (TSS) of the NDRG1 gene, Figure 2A. As shown in Figure 2B and 2C, MYCN and LSD1 were both recruited selectively at the transcriptional start site (TSS) of the NDRG1 gene but not at distal sites (−10kb), indicating that the MYCN/LSD1 complex binds to the NDRG1 promoter. We find also that MYCN binding was unaffected by TCP or LSD1 depletion implying that MYCN binding does not require LSD1 while, in contrast, LSD1 binding was reduced in TCP-treated and LSD1-KD samples, suggesting that the binding of LSD1 require the catalytic activity of the enzyme. Next, we monitored the histone modifications occurring at NDRG1 promoter (Figure 2D, 2E). Depletion of LSD1 enhances H3-acetylation whereas it reduces the repressive mark H3K27me3, consistent with the induction of NDRG1 expression in these cells. Overall, our findings demonstrate that: 1) both LSD1 and MYCN bare recruited to the NDRG1 promoter chromatin to repress NDRG1 expression; 2) LSD1 inhibition is sufficient to relieve MYCN-driven NDRG1 repression.

### Effects of TCP and SP2509 inhibitors on LSD1/MYCN-mediated regulation of NDRG1

**Figure 3:**
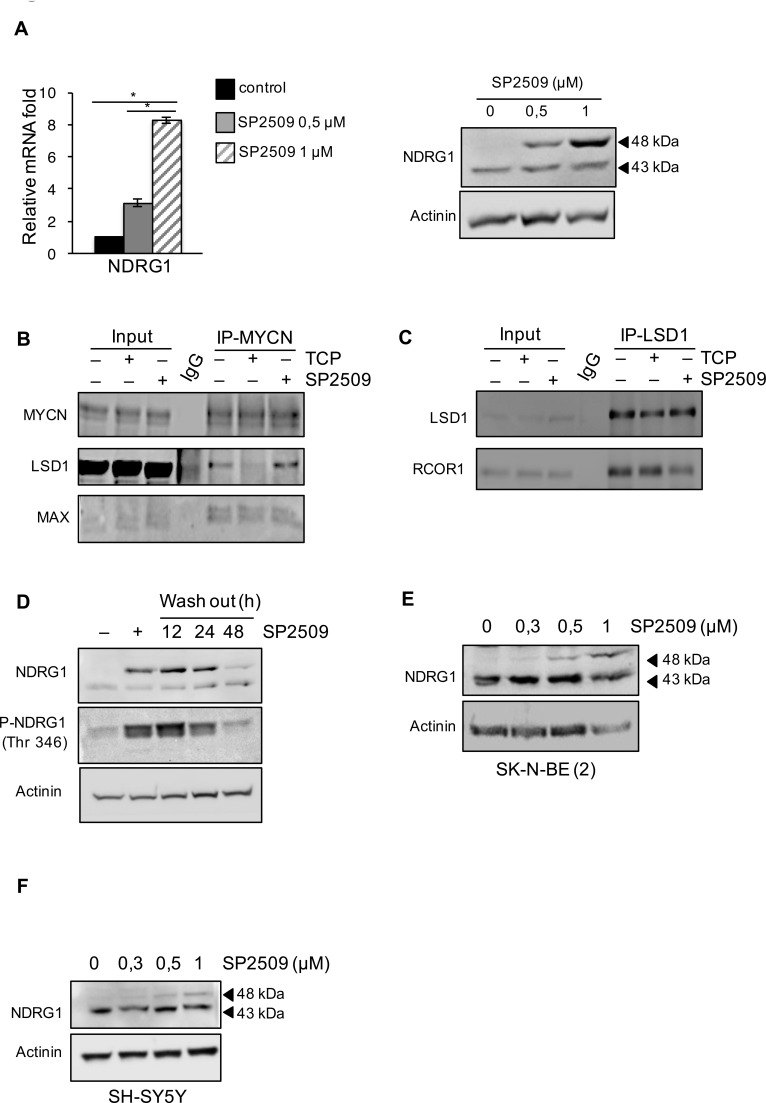
A. NDRG1 gene expression was determined by qRT-PCR or by western blot in Tet-21/N cells treated with SP2509 at different concentrations, as indicated. *, statistical significance (*P* < 0.01; Student t test) **B**. Co-Immunoprecipitation with MYCN antibody was performed in 293T cells co-transfected with LSD1 and MYCN expression vectors and treated with TCP, SP2509 or vehicle. Extract were analyzed by western blotting with MYCN, LSD1 and MAX antibodies as indicated. **C**. Interaction between endogenous LSD1 and MYCN in Tet-21/N cells, treated with TCP, SP2509 or vehicle, was assessed by co-Immunoprecipitation. Cell lysates were immune-precipitated with a LSD1 antibody Western blot analysis was performed on immuno-precipitated extracts with LSD1 and RCOR1 antibodies. IgG-sample was used as negative control. **D**. Tet-21/N cells were treated for 48 h with SP2509 or vehicle and then released into fresh medium for the indicated times. Cellular extracts were prepared and stained with anti-NDRG1 and phospho-NDRG1 (Thr 346). **E**. and **F**. Cell extracts from SK-N-BE (2) and SH-SY5Y cells treated with SP2509 at the indicated concentrations were prepared and probed with NDRG1 antibody. Actinin was probed as loading control.

During last years several small molecular inhibitors of LSD1 based on different molecular mechanisms have been developed [33]. SP2509 is a reversible inhibitor of LSD1 and differently from TCP does not target the catalytic activity of the enzyme. SP2509 attenuates the binding of LSD1 to CoREST and it has been found to be effective in inhibition of cultured and primary AML blasts [34]. To further substantiate the role of LSD1 in the suppression of NDRG1 we analyzed the effects of treatment of NB cells on NDRG1 expression by treatment with this different LSD1 inhibitor. As shown in Figure 3A, SP2509 treatment enhances NDRG1 mRNA expression and increases the NDRG1 48kDa protein levels in a dose dependent manner. Thus, both TCP and SP2509 enhance NDRG1 expression albeit these drugs inhibit LSD1 through different mechanisms. Because LSD1/MYCN negatively controls NDRG1 transcription we assessed whether TCP or SP2509 may interfere with the LSD1/MYCN interaction. To this end, HEK293T cells co-transfected with expression vectors encoding LSD1 and MYCN were exposed to TCP and the complex between MYCN and LSD1 was analyzed by immunoprecipitation. As shown in Figure 3B, LSD1 and MYCN readily interact in the absence of TCP but their association was impaired in presence of the drug. This inhibitory effect of TCP is specific to LSD1-MYCN complex since it did not interfere with the interaction of MYCN with its endogenous partner MAX (Figure 3B). In contrast, SP2509 did not inhibit the interaction between LSD1 and MYCN. Also LSD1/CoREST association was inhibited by SP2509, not by TCP (Figure 3C). Collectively these results demonstrate that LSD1 activity is necessary for the interaction with MYCN, not with CoREST. Thus, inhibition by TCP or SP2509, de-represses NDRG1 expression, albeit the two drugs have a marked different mode of action.

Since SP2509 is a reversible inhibitor of LSD1, we tested whether re-activation of NDRG1 by SP2509 treatment was reversible. Tet-21/N cells were treated with SP2509 for 48 hrs and then cells were washed, fed with normal medium and collected at 12, 24 and 48 hrs thereafter SP2509 wash out. Results in Figure 3D shows that NDRG1 expression decreases in a time dependent manner following removal of the SP2509, demonstrating that NDRG1 activation is directly dependent upon LSD1 inhibition.

To address if LSD1 inhibition affects NDRG1 expression in the context of MYCN amplification, we analyzed the effect of SP2509 in a non-amplified MYCN SH-SY5Y cell line. Moreover, since activation of NDRG1 may also occurs as result of p53 binding in colon cancer cell lines [35] we also used the p53 mutated, MYCN-amplified NB cell line SK-N-BE (2) to address the relative contribution of p53 in NDRG1 activation. The SH-SY5Y (MYCN non-amplified) and MYCN-amplified p53 mutated SK-N-BE (2) cells were treated with SP2509 at different concentration for 48 hrs and western blot was performed using the NDRG1 antibody. Results reported in Figure 3E, 3F show that up regulation of the 48 kDa NDRG1 band is observed in both cell lines demonstrating that NDRG1 activation by LSD1 inhibition is not due to p53 activity and is not cell specific.

Collectively our results demonstrate that NDRG1 expression is modulated by LSD1 and that pharmacological LSD1 inhibition in NB cells up-regulates NDRG1 expression.

### Effect of LSD1 inhibition on migration and invasion of NB cells

NDRG1 over-expression promotes formation of adherent junctions and inhibits cell migration and invasion in several types of tumors cells indicating that NDRG1 inhibits the establishment of the epithelial-mesenchymal transition (EMT) program [18, 36]. Our findings suggest that LSD1 pharmacological silencing might control EMT in NB tumor cell lines by upregulating NDRG1 expression.

**Figure 4:**
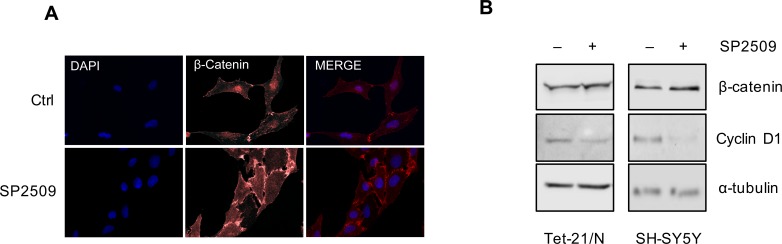
A. Tet-21/N cells were treated with SP2509 or vehicle, fixed and processed for anti-β-catenin immunofluorescence and DAPI staining **B**. Western blot assay of protein extracts of Tet-21/N and SH-SY5Y cells treated as indicated using β-catenin and Cyclin D1 antibodies. α-tubulin has been probed as loading control. *, *P* < 0,01.

LSD1 was demonstrated to activate the Wnt/β-catenin signaling pathway by down-regulating the pathway antagonist DKK1 in colorectal cancer cells [37]. In different studies NDRG1 overexpression has been shown to inhibit β-catenin phosphorylation inducing its accumulation at cell membranes [21]. We examined if NDRG1 activation mediated by pharmacological inhibition of LSD1 affected β-catenin subcellular localization. To this end we performed immunofluorescence to detect β-catenin in Tet-21/N cells untreated (Ctrl) or treated with SP2509. As shown in Figure 4A, SP2509 enhanced β-catenin accumulation on cellular membrane. A modest increase of β-catenin protein levels was detected in Tet-21N and SH-SY5Y cells by immuno-blotting, Figure 4B, suggesting that SP2509 treatment enhanced β-catenin accumulation on cellular membrane. Consistent with such effect, expression of the β-catenin downstream target, Cyclin D1 was down-regulated in LSD1 inhibited cells. These results indicate that pharmacological treatment of NB cells with LSD1 inhibitor results in NDRG1 activation and suggest that the anti-metastatic activity of NDRG1 in NB occurs at least in part through accumulation of β-catenin at cell membrane.

**Figure 5:**
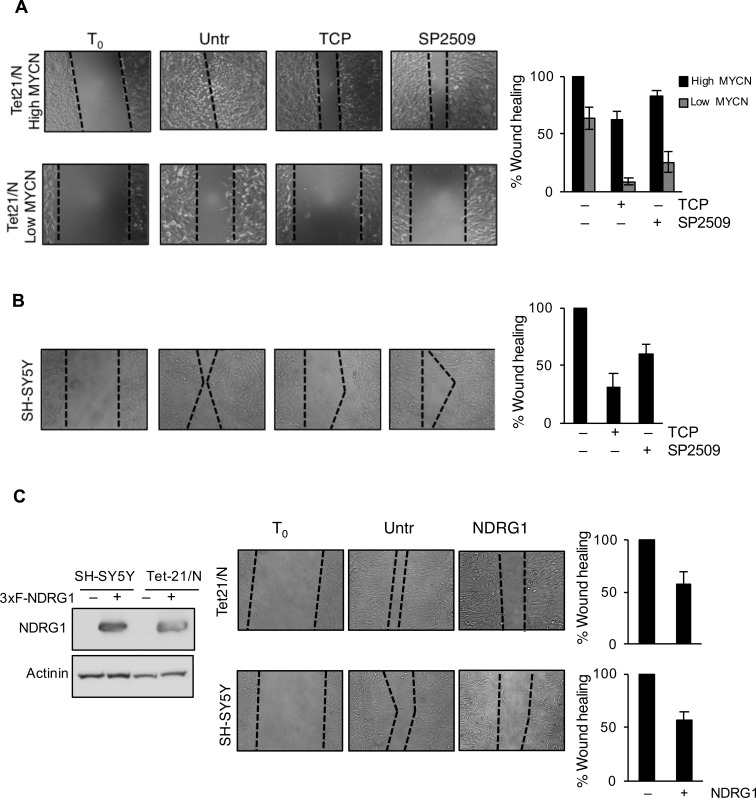
LSD1 inhibition reduces migration of Neurobastoma cells **A**. Wound healing of Tet-21/N (High MYCN), Tetracycline-treated Tet-21/N (Low MYCN) and **B**. SH-SY5Y cells treated with vehicle, TCP or SP2509. **C**. Wound healing was performed in Tet-21/N and SH-SY5Y cells 3XFlag-NDRG1 or mock transfected. Migration was assessed under treatment conditions at several time points using a scratch wound assay. Representative phase contrast images were shown acquired at 24hrs after scratch. Western blot shows NDRG1 protein levels in 3xFlag-NDRG1 or mock transfected Tet-21/N and SH-SY5Y cells. Actinin was used as loading control. Graphs showing the results represent the mean ± SD of three independent experiments carried out in duplicate. Statistical significance *P* < 0,01.

**Figure 6:**
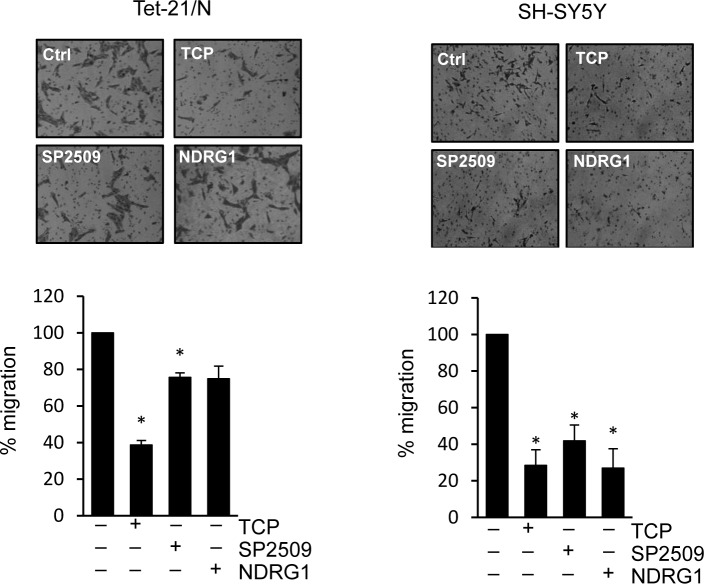
Trans-membrane migration assay of Tet-21/N and SH-SY5Y cells treated with vehicle, TCP or SP2509, or NDRG1-transfected Graphs showing the results represent the mean ± SD of three independent experiments carried out in duplicate. *, *P* < 0,05.

We then asked whether treatment with LSD1 inhibitors and over-expression of NDRG1 might impair the migration and invasion of tumor NB cell lines. Untreated Tet-21/N (High MYCN), tetracycline-treated (Low MYCN) and the SH-SY5Y cells were used in wound-healing assays in presence or absence of TCP or SP2509. Both Tet-21/N (High MYCN) and SH-SY5Y cells filled almost completely the wounded area 24hrs after scratching the cell monolayer, while Tet-21/N (Low MYCN) showed impaired migration efficiency (Figure 5A and 5B). TCP or SP2509 treatment markedly suppressed repair of the wound area. Such inhibitory effect was enhanced in Low-MYCN cells suggesting that reduction of MYCN levels cooperates with LSD1 in blocking the migration of LSD1-KD cells. Next, we tested the effect of NDRG1 over-expression on cell invasiveness of Tet-21/N and SH-SY5Y cells. Both cell lines were transfected with a human expression vector for NDRG1, whose expression was assayed by Western blots, Figure 5C. We determined that overexpression of NDRG1 recapitulates the inhibitory effects exerted by LSD1 inhibitors. Next, we determined the effect of LSD1 inhibition and NDRG1 over-expression on cell invasion (Figure 6). Using the trans-well migration assay, we showed that NDRG1 overexpression as well as LSD1 pharmacological inhibition in both Tet-21/N and SH-SY5Y cells resulted in a significant reduction ( ≥ 25%) of migratory capacity compared with control cells.

These findings demonstrated that pharmacological inhibition of LSD1 blocks migration and invasion of neuroblastoma cells and most importantly overexpression of NDRG1 recapitulate these effects. Collectively, these findings demonstrated that pharmacological inhibition of LSD1 suppresses the mobility and invasiveness of cancer cells through up-regulation of NDRG1.

### NDRG1 expression during differentiation and in NB tumors

**Figure 7:**
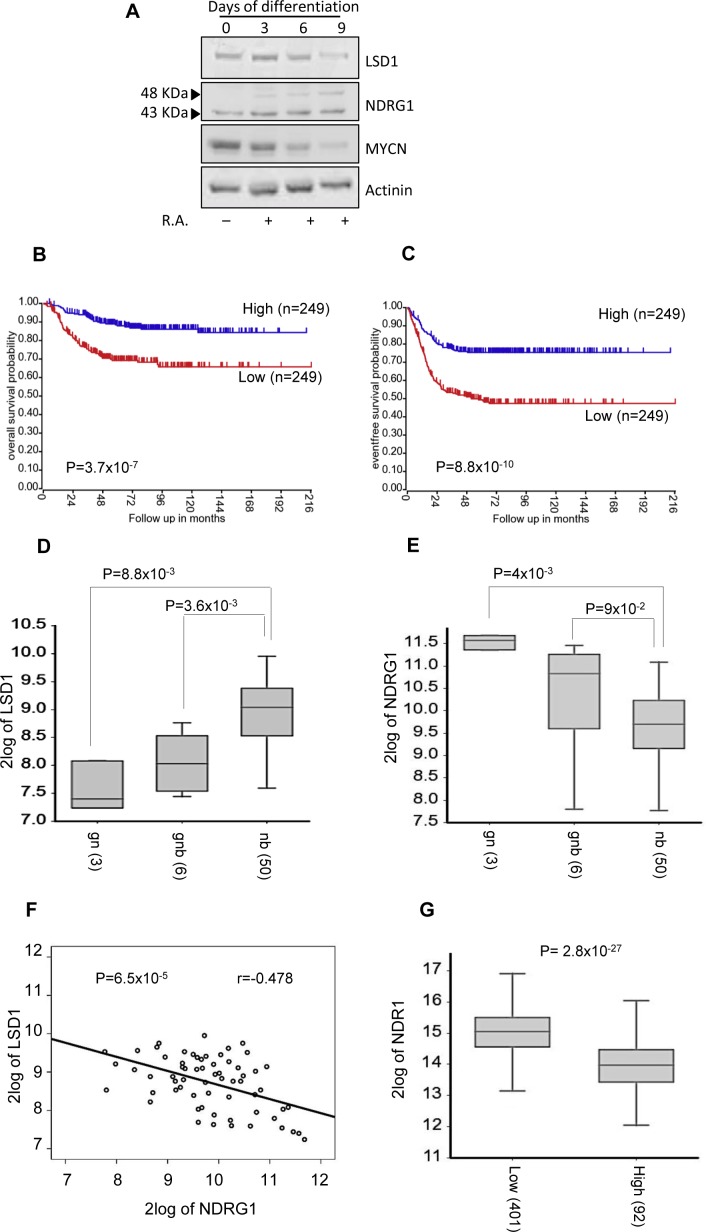
A. SK-N-BE (2) cells were treated with RA up to 9 days. LSD1, NDRG1 and MYCN protein levels were detected in differentiated SK-N-BE (2) cells at the indicated days by western blotting NDRG1 expression is associated with good outcome and differentiated tumors. **B**. and **C**. Low NDRG1 expression is associated with negative prognosis. The number of tumors is indicated in parentheses. Kaplan-Meier analysis is shown, with individuals grouped by median of expression of NDRG1. Log-rank P values are shown. Changes in expression for LSD1 **D**. and NDRG1 **E**. in ganglioneuroblastoma (GNB), ganglioneuroma (GN) and neuroblastoma (NB). **F**. Inverse correlation between the expression values of NDRG1 and LSD1 in NB tumors (Pearson's correlation coefficient is shown). **G**. Box plot showing differential NDRG1 expression in NB tumors without (Low) or with (High) MYCN amplification.

It had been shown that LSD1 expression is reduced following *in vitro* induced differentiation of neuroblastoma cells [14, 38]. The findings reported above indicated that high levels of LSD1 inversely correlate to NDRG1 expression. To address the relative expression levels of MYCN, LSD1 and NDRG1 during differentiation, SK-N-BE(2) cells were induced to differentiate by treatment with RA. Cell samples were collected at different time points after treatment and analyzed for LSD1 and NDRG1 and MYCN expression levels. As shown in Figure 7A, *in vitro* induced differentiation results in reduction of LSD1 and MYCN expression along to a concomitant up-regulation of NDRG1 levels. These results further confirm the role of LSD1 on NDRG1 expression and highlight their antagonism during differentiation of NB cells. Moreover these data strongly suggest that NDRG1 can be used as marker of neuroblastoma differentiation *in vivo*.

To further corroborate the mutually exclusive expression of NDRG1 and LSD1 we examined the relevance of NDRG1 in neuroblastoma patients. Independent studies have shown that low NDRG1 levels are associated with worse prognosis for patients with breast, glioma, colorectal, esophageal squamous cell carcinoma, and prostate cancer [19]. More recently, it has been reported that low levels of NDRG1 is associated with poor prognosis in neuroblastoma patients [39]. In sharp contrast, LSD1 expression inversely correlates with differentiation and adverse outcome [14, 38] of neuroblastoma. Our *in vitro* findings imply that also in patients high LSD1 and low NDRG1 levels should be inversely correlated in metastatic Neuroblastomas. To this end we analyzed available RNAseq data of 498 NBs and we found that high NDRG1 expression correlates with better overall and event-free survival (Figure 7B and 7C, Mann-Whitney test, *P* = 3.7×10^−7^ and *P* = 8.8×10^−10^). Next, we analyzed LSD1 and NDRG1 expression in a microarray gene expression data of 59 NBs, of which 50 were neuroblastoma and 9 were ganglioblastoma and ganglioneuromas. LSD1 expression was considerably higher in neuroblastoma than in ganglioblastomas and ganglioneuromas (Figure 7D). In contrast, NDRG1 expression was higher in well-differentiated tumors (Figure 7E). Thus, LSD1 and NDRG1 appear to be expressed in opposite fashion in NB. Accordingly, we found that the expression of NDRG1 is inversely correlated with the expression of LSD1 (Figure 7F, *P* = 6.5×10^−5^). Finally, we determined that NDRG1 expression levels were appreciably lower in MYCN-amplified NB samples (Figure 7G). Collectively, these findings demonstrated that high levels of LSD1 and NDRG1 expression are mutually exclusive in neuroblastoma, and the expression levels of NDRG1 are significantly lower in MYCN-amplified tumors.

## DISCUSSION

In the current study, we demonstrated that LSD1 in cooperation with MYCN controls cell migration and invasiveness of neuroblastoma cells through transcription regulation of the metastatic suppressor NDRG1. Our findings support a previously unidentified model to control EMT in neuroblastoma, proposing that epigenetics changes caused by LSD1 inhibition lead to up-regulation of NDRG1 thereby inducing an NDRG1-dependent inhibitory effect on cell migration and invasiveness of neuroblastoma cells

We found that in neuroblastoma cells the MYCN/LSD1 complex binds and represses NDRG1 expression. Following LSD1 inhibition epigenetics changes occur on the chromatin region surrounding the transcriptional start site of NDRG1 leading to transcription activation of NDRG1 gene expression. In a recent study it has been shown that the signal cascade mTORC2/serum glucocorticoid induced protein kinase1 (SGK1) phosphorylates NDRG1 [20]. It is likely that LSD1-KD may also affect mTORC2/GSK1 pathway, clearly further investigations are required to clarify the role of LSD1 in the phosphorylation of NDRG1.

LSD1 inhibition suppresses motility and invasiveness of NB cells and ectopic over expression of NDRG1 phenocopy the pharmacological treatments with LSD1 inhibitors, suggesting that de-repression of NDRG1 expression plays a causative role in blocking cell migration and invasiveness. Moreover, lowering MYCN expression we observed a cooperative inhibition with TCP to restrain cell mobility, suggesting that MYCN and LSD1 cooperatively control EMT.

High-risk neuroblastoma (NB) with MYCN amplification is a highly metastatic tumor in children. NB presenting with hematogenesis metastasis is one of the most difficult cancers to cure [1, 40]. EMT is an important process that contributes to tumor invasion and dissemination [41]. How MYC control EMT is largely unknown [42]. EMT process requires extensive reorganization of the epigenetic information of the cells. Previous works showed that SNAIL represses transcription of epithelial genes such as E-cadherin, by recruiting repressive chromatin-modifying factors including Polycomb repressive complex 2 and LSD1-CoREST complex [41]. Our findings of targeting NDRG1 expression through LSD1 inhibitors add new insight on how MYCN may control EMT. Thus, LSD1 controls EMT through at least two different mechanisms, as co-factors of SNAI1 function and in association with MYCN as a direct epigenetic regulator of NDRG1 expression. Previous work showed that blocking interactions of LSD1 with SNAI1 blocks NB cell invasion [43]. The findings reported here add further support to the critical role of LSD1 in EMT and most importantly highlight an additional mechanism through which LSD1 inhibition affects cell migration and invasiveness of NB cancer cells. Clearly multiple signaling pathways cooperate in the initiation and progression of EMT and cooperation between different pathways likely occurs in a synergistic manner and in a cell-type specific fashion.

Therapy for high-risk patients includes differentiating agents. Previous studies showed that NDRG1 expression is regulated by differentiation-related environments [19]. We determined that during RA-mediated *in vitro* differentiation of NB cells the NDRG1 protein increases during time and inversely correlates with LSD1 and MYCN protein expression. Thus, these data address that NDRG1 is a biologically important MYCN/LSD1 target, and it is inversely expressed in relation to MYCN and LSD1 during NB differentiation.

The relative expression levels of NDRG1, LSD1 and MYCN were further analyzed in neuroblastoma patients. Analysis of publicly available expression data of large number of NBs highlighted that high NDRG1 expression correlates with better overall and event-free survival. Interestingly, high levels of LSD1 and NDRG1 expression are mutually exclusive in neuroblastoma tumors and NDRG1 expression levels are significantly lower in MYCN-amplified NB samples. Collectively, these findings support and corroborate the broad significance of our *in vitro* results, and suggest that NDRG1 and LSD1 expressions can be considered as valuable biomarkers to monitor NB development in humans.

In summary, our findings uncover a previously unidentified model in the control of EMT, suggesting that MYCN/LSD1 inhibition de-represses NDRG1 expression, thereby inducing an NDRG1-dependent inhibitory effect on cell migration and invasiveness of neuroblastoma cells. These findings raise the possibility that improved approaches aimed to target the epigenetic control of NDRG1 expression may lead to development of novel strategies to inhibit the invasive potential of neuroblastoma cells.

## MATERIALS AND METHODS

### Cell culture and treatments

Human HEK 293T, SH-SY5Y and SHEP Tet-21/N cells were cultured in Dulbecco's modified Eagle's Medium (DMEM) supplemented with antibiotics, 10% fetal calf serum. SK-N-BE (2) was cultured in 1:1 mixture DMEM/F-12 containing 10% FBS. All cell lines were incubated at 37°C in humidified atmosphere with 5% CO_2_. Tet-21/N cells are cultivated with (Low MYCN) or without (High MYCN) tetracycline (6 days). When indicated, cells were treated with TCP (1mM, Enzo Life Sciences), SP2509 (0,3/0,5/1 μM, Cayman Chemical Company) or 10054-F4 (75 μM, Sigma) for 24 or 48 hrs. To induce differentiation in SK-N-BE (2) cells were exposed to 10 μM all-*trans* Retinoic Acid for 9 days.

### LSD1 Knock-Down

100 nM siRNA targeting LSD1 (GE Dharmacon) or scramble were transfected in Tet-21/N cells using a MicroPorator Digital Bio Technology, according to the recently described protocol [6]. Briefly, 2×10^6^ cells were collected by trypsin/EDTA digestion, washed once with calcium and magnesium-free PBS and resuspended in 100 μl of resuspension buffer, mixed with siRNA or scramble and electroporatated according to the manufacturer's protocol. Transfected cells were seeded in a 100 mm dish in antibiotic-free DMEM supplemented with 10% FBS. The efficiency of siRNA to knockdown LSD1 protein expression was assayed 48h after transfection by western blot.

### RNA sequencing

RNA was prepared from Tet-21N cells treated with TCP or with siLSD1 and control untreated cells. RNA-seq libraries (two biological replicas for each sample) were generated using TruSeq RNA Sample Prep Kit v2 (Illumina) according to the manufacturer's recommendation. All the high-throughput sequencing experiments were run on a NexSeq 500 (Illumina) sequencer at the Genomix4life S.R.L., Baronissi, Salerno, Italy, according to standard operating procedures. Raw sequences files (−fastaq files) were aligned to the human genome (h19 version), gene-level quantification was performed using R-SEM and UCSC annotation [44]. Subsequently, data were normalized with VOOM method [45] and differential expression evaluated with limma Bioconductor packages. Differential expressed genes were detected applying the following cutoff: log2 Fold Change ≥ 1 and FDR ≤ 0.1. RNA-seq data were deposited to NCBI GEO and are available under accession number GSE80753.

### RNA extraction and qRT-PCR

RNA was extracted from NB cells using EuroGold Trifast (EuroClone). cDNA was generated using Quantitec Reverse Transcription Kit (Qiagen), according to manufacturer's protocol. Quantitative analysis was performed using SYBR Green 2X PCR Master Mix (Applied Biosystem). Each sample was run in triplicate and normalized to the expression of housekeeping beta-glucoronidase (GUSb) gene as previously described [6]. Primers are presented in Supplementary Table S1.

### Protein extraction and western blot

Whole-cell extracts were obtained using buffer F (10 mM TrisHCl pH 7.5, 150 mM NaCl, 30 mM Na4O7P2, 50 mM NaF, 5 mM ZnCl2, 0.1 mM Na3VO4, 1% Triton, 0.1mM PMSF). 50 μg of protein extracts were loaded and separated by SDS-PAGE and WB was performed with indicated antibodies. For NDRG1 silencing in Tet-21/N cells, 3 μg/10^6^ cells of shRNA plasmid (Santa Cruz) targeting NDRG1 was used with the protocol described above.

### Chromatin immunoprecipitation assay

Chromatin immunoprecipitation assays were performed as recently described [6]. Briefly 1×10^7^ cells were cross-linked using formaldehyde to a final concentration of 1% and reaction was stopped using 0.125M Glycine. Cell pellet was resuspended in Cell Lysis Buffer and after 6000 rpm centrifugation RIPA buffer were added to perform nuclei lysis. DNA shearing was conducted by sonication using Bioruptor (Diagenode). A small aliquot of sonicated material was put aside and remaining sample immunoprecipitated using 5 micrograms of ChIP-grade antibodies. Rec-sepharose Protein A or G beads (Invitrogen) were used to immobilize immuno-complexes and after RNAse-A treatment (37°C 1 hour) reverse cross-linking were performed using Proteinase K (Roche) for 6 hours at 65°C. Immunoprecipitated DNA was purified using Phenol/Chloroform and Ethanol precipitation techniques. The antibodies used are listed in Table S2. The immunoprecipitated DNA was quantified by qPCR with the primer sets described in Table S1.

### Migration assays

In migration experiments, 2,5 μg/10^6^ cells of 3xFLAG-NDRG1 or empty vector were transiently transfected into Tet-21/N by electroporation, by protocol as described previously. For transient transfections of SH-SY5Y, cells cultured on 100 mm dishes were transfected with 3xFLAG-NDRG1 plasmid or empty vector using Lipofectamine 2000 according to manufacturer's protocol. The expression of protein was determined by western blot. For the wound-healing assay, NDRG1-trasfected or control cells were plated to confluence in a 12-well plate and scraped with a p200 pipet tip to create a scratch of the cell monolayer; when indicated cells were treated with TCP or SP2509 for an overnight before scratch and during the whole experiment. Cells were then allowed to fill the wounded area for 2 days and images were acquired using a Nikon Eclipse TE 2000-U microscope. Percentage of wound healing was measured as following: [(empty area at T_0_)-(empty area at 24hrs)]/(empty area at T_0_) x 100. For trans-membrane migration assay, cells were NDRG1-transfected or pre-treated with TCP or SP2509 for an overnight, before plating (150000 cells/chamber) in free serum medium in the upper side of chambers (BD Falcon Cell Culture Inserts). In the wells 20% of FBS was used as chemo-attractant. After 24 h, non-migrating cells were scraped-off, whereas migrating cells were stained with a 20% ethanol-1% crystal violet solution for 10’, washed thrice with water and counted at least in ten fields with a 10x objective. For each assay three independent experiments were carried out in duplicate.

### Co-immunoprecipitation

Co-immunoprecipitation assays were performed using Tet-21/N and HEK 293T cells. 293T cells were transiently co-transfected with 3xFLAG-LSD1, 3xFLAG-MYCN or scramble by the polyethylenimine (PEI 25 K) method. 1 mg of protein extract from Tet-21/N cells or 0,3 mg from HEK 293T cells, treated with TCP, SP2509 or vehicle, were incubated respectively with LSD1 or MYCN antibody and processed as previously described [6, 46]. Protein interactions were assessed by immunoblotting using the indicated antibodies.

### Immunofluorence

For immunofluorescences assay Tet-21/N were seeded on coverslips and treated as indicated. Cells were than fixed in 4% paraformaldehyde in PBS, permeabilized in 0.1% Triton X-100 in PBS, pre-blocked in 2% BSA- 3%NS-PBS for 30 min at room temperature, and then incubated for 1 h at 37°C with mouse anti-β-catenin. Primary antibodies were detected by incubation with Cy3-coniugated anti-mouse. Images were acquired using a Nikon Eclipse TE 2000-U microscope.

### Gene expression data for survival analysis and association with neuroblastoma stages

Normalized gene expression data from RNA sequencing of 498 tumors were downloaded from “R2: Genomics Analysis and Visualization Platform” (GEO ID: GSE62564). To test association of gene expression levels with overall survival and event free survival, individual gene expression profiles were dichotomized by median split into ‘high’ or ‘low’ expression groups, and Kaplan-Meier survival curves were plotted for each group. Long rank test was used to evaluate the significant difference between the two groups. Another set of gene expression data of 64 tumors (GEO ID: GSE12460) including 50 NB, 6 ganglioneuroblastoma and 3 ganglioneuroma was downloaded. Mann-Whitney test was used to test the significant different gene expression among groups. The correlation between the gene expression between NDRG1 and KDM1A was evaluated by Pearson correlation in 64 NBs. The gene expression data for Low and High MYCN expression (493 samples) were generate by customized 4×44K oligonucleotide microarrays produced by Agilent Technologies and analyzed as previously reported [47].

### Statistical analysis

All experiments were repeated two or three times. Graphs representing data express mean ± SD. Statistical significance was obtained by unpaired, two-tailed Student *t* test. *P* < 0,05 was considered statistically significant.

## SUPPLEMENTARY MATERIALS




