# Supplementary material for: LSD1 mediates MYCN control of epithelial-mesenchymal transition through silencing of metastatic suppressor NDRG1 gene
Source: Oncotarget. 2016 Nov 22;8(3):3854–69. doi: 10.18632/oncotarget.12924 (PMC5354800; doi:10.18632/oncotarget.12924)
Supplement: Supplementary file 1 [file oncotarget-08-3854-s001.pdf]

LSD1 mediates MYCN control of epithelial-mesenchymal transition through silencing of metastatic suppressor NDRG1 gene

Supplementary Material

Table S1: oligos used in this work

	Gene	FW	Rev
qChIP	NDRG1 (TSS)	CGGATGGTGAACTGACGA	CCCGGACGTAAACAAACCT
qChIP	NDRG1 (-10Kb)	CCCATTGCTTTAAATCTGCTCT	TCAAAGCACTCTCTCGCTTTT
qRT-PCR	NDRG1	GGGCTGAAAAGCATTATTGG	CTCCACCATCTCAGGGTTGT
qRT-PCR	Desmoplakin	GCTAAACGCCGCCAGGAT	CCGCATGACTGTGTTGGAAT
qRT-PCR	Vimentin	CCAGCCGCAGCCTCTACG	GCGAGAAGTCCACCGAGTCC
qRT-PCR	Occludin	GCCGGTTCCTGAAGTGGTT	CGAGGCTGCCTGAAGTCATC
qRT-PCR	N-cadherin	CTGTGGGAATCCGACGAATGG	GTCATTGTCAGCCGCTTTAAGG
qRT-PCR	E-cadherin	CCGCTGGCGTCTGTAGGAAGG	GGCTCTTTGACCACCGCTCTCC
qRT-PCR	Smooth muscle α-actin	CACTGCCGCATCCTCATCCT	TCATGATGCTGTTGTAGGTGGT
qRT-PCR	GUSb	GTGGGCATTGTGCTACCTC	ATTTTTGTCCCGGCGAAC

Table S2: antibodies used in this work

Protein	Used for	Manufacturer
Actinin	WB	sc-17829, Santa Cruz
NDRG1	WB	ab37897, Abcam
P-NDRG1 (Thr 346)	WB	#3217, Cell Signaling
LSD1	ChIP, co-IP, WB	ab17721, Abcam
MYCN	ChIP, co-IP, WB	sc-53993, Santa Cruz
H3Ac	ChIP	06-599, Millipore
H3K27me3	ChIP	07-449, Millipore
β-Catenin	WB, IF	610153, BD Biosciences
CyclinD1	WB	sc-8396, Santa Cruz
α-Tubulin	WB	T5168, Sigma-aldrich
RCOR1	WB	#07-455, Upstate
Max	WB	sc-197, Santa Cruz

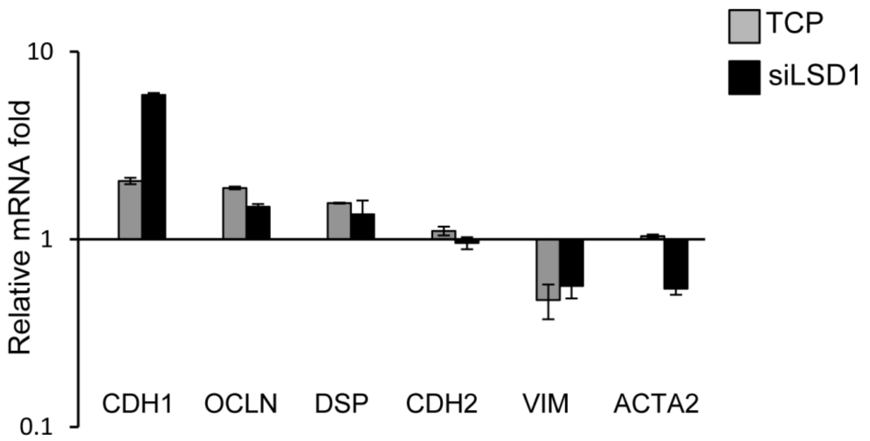

Supplementary Fig 1.EMT markers mRNAs expression analysis after TCP treatment and LSD1-KD of Tet-21/N cells through quantitative RT-PCR. Expression profiles were normalized against control cells. Data represent the mean ± SD of three independent experiments. Changes in mRNA expression are all statistically significant ($p<0,05$; Student t test).

Fig. 1D

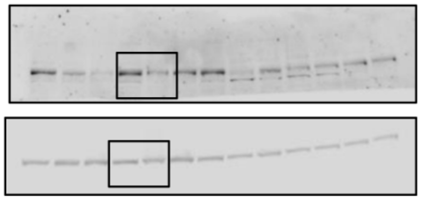

Fig. 1E

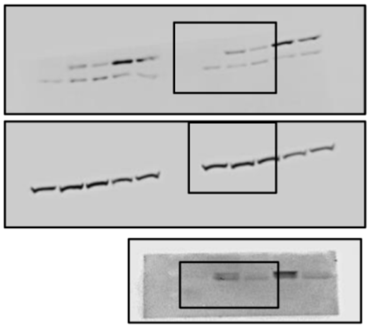

Fig. 1F

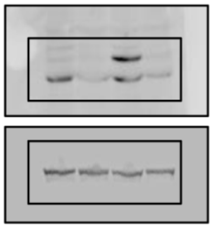

Fig. 1G

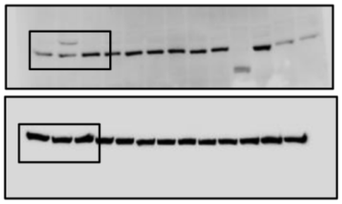

Fig. 3A

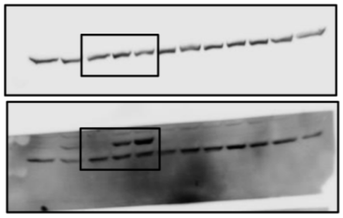

Fig. 3B

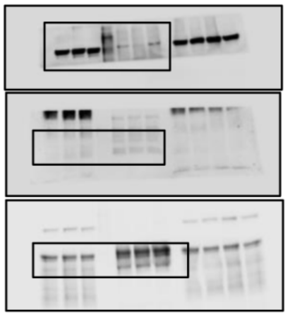

Fig. 3C

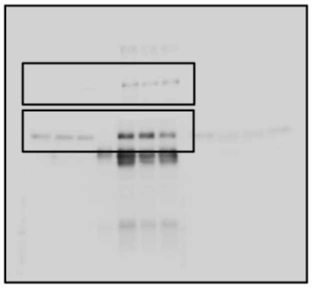

Fig. 3D

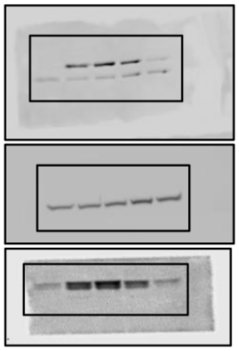

Fig. 3E

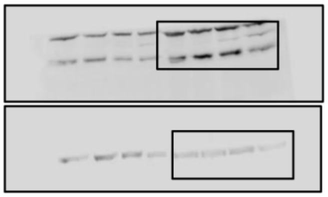

Fig. 3F

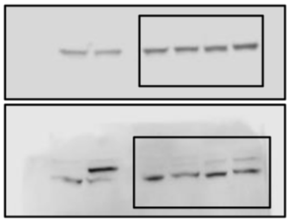

Fig. 4B

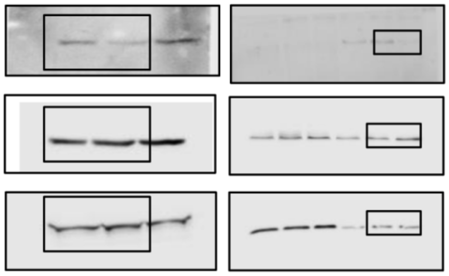

Fig. 5B

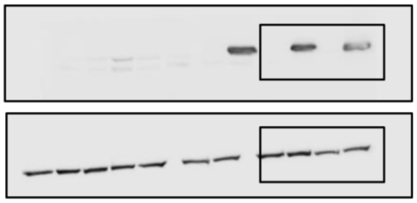

Fig. 7A

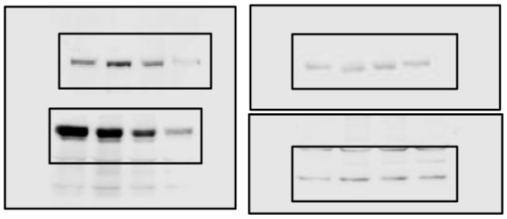

Supplementary Fig. 2: Full scans of Western data
